# The Pathological Anatomy of the Human Body

**Published:** 1846-10

**Authors:** 


### ART. IV.

1. *Pathologische Anatomie des menschlichen Körpers.* Von JULIUS VOGEL. Erste Abtheilung. (Allgemeiner Theil.)—*Leipzig,* 1845.

*The Pathological Anatomy of the Human Body.* By JULIUS VOGEL.—*Leipsic,* 1845. pp. 533.

2. JULII VOGEL *Icones Histologiæ Pathologicæ. Tabulæ Histologiam Pathologicam illustrantes. Viginti sex Tabulæ, continentes* CCXCI *Figuras, quarum* CCLXX *ad naturam delineatæ sunt.*

*Erläuterungstafeln zur pathologischen Histologie mit vorzüglicher Rücksicht auf sein Handbuch der pathologischen Anatomie,* herausgegeben von Dr. JULIUS VOGEL, ausserordentl. Professor der Medizin in Göttingen. *Sechs und zwanzig Tafeln mit* 291 *Figuren, wovon* 270 *nach der Natur gezeichnet sind.*—*Leipzig,* 1843.

*Illustrations of Pathological Histology, illustrative of his Manual of Pathological Anatomy.* By Dr. JULIUS VOGEL, Extraordinary Professor of Medicine at Gottingen. *Twenty-six plates, with* 291 *Figures, of which* 270 *are drawn from Nature.*—*Leipsic,* 1843. pp. 120.

DR. JULIUS VOGEL has long been known as an accurate chemist, a good microscopic observer, and a sound pathologist,—qualifications which eminently fit him for the duty he has now undertaken of publishing a treatise on morbid anatomy. In addition to the works whose titles head this article, he is favorably known as the author of essays "on the Sputa in various Diseases," and on "the Physiology and Pathology of Pus," of the chemical portion of Wagner's 'Elements of Physiology,' of an "Introduction to the use of the Microscope in the Chemical Analysis of Animal Matters" (reviewed in Vol. XVII, p. 424), and of the articles "Inflammation," "Morbid Tissues," and "Hypertrophy," in Wagner's 'Cyclopædia of Physiology,' now in the course of publication.

In the introduction to the present work we find some excellent remarks on the proper means of acquiring a knowledge of pathological anatomy, and as we believe that these means are neglected, and indeed, despised, by some of our English pathologists, we avail ourselves of this opportunity of pointing out to them what are deemed by their German brethren the qualifications requisite for the successful cultivation of this department of science.

"The study of those morbid changes to which the various parts of the body are liable, depends on a thorough previous knowledge of their normal relations; hence pathological anatomy requires a perfect knowledge of the structure of the healthy body, and of the uses of its different parts, in order to be able to estimate the influence which any morbid alteration of an organ impresses on its function. We must not content ourselves with studying the coarser changes, such as are visible to the naked eye, but we must examine those finer modifications of the tissues which can be recognized only by the microscope; hence the necessity for an accurate knowledge of general anatomy or histology. In the investigation of delicate points connected with pathological histology the microscope is altogether indispensable, and the application of chemical reagents must be observed under it. Chemical analysis is indeed of the greatest importance in pathological anatomy, being the only means by which we can on several points obtain the desired information." (p. xxxii.)

The whole work is divided into two parts,—the general and special. Of these we have at present only the former, the latter being promised in the course of the present year. The Icones, which were published about three years ago, refer both to the special and general department, there being twelve plates illustrative of general, and fourteen of special pathological anatomy.

GASES. The first chapter is devoted to the consideration of the *abnormal development of gaseous matters.* Accumulations of gas within the body may arise from very different causes.

1. *They may be produced by the external pressure of the atmospheric air.* The mechanism of this form of origin is most strikingly seen in those cases of general emphysema which arise from an injury to the lungs dependent on a penetrating wound of the thorax. Our author gives the following graphic sketch of the progress of the affection.

"The air admitted into the cellular tissue of the thorax gradually works its way over the body, and the emphysema thus becomes more or less general. The orbits become closed up; the eyes and mouth remain shut, in consequence of the swollen condition of the eyelids and lips; the nose is hidden between the tumid cheeks; the skin of the neck is so monstrously distended that all distinction between the head and neck disappears; the scrotum swells to such a size as to conceal the penis; the limbs enlarge and assume a cylindrical form; the palms of the hands and the soles of the feet (in consequence of their firm connexion with the subjacent tissues) being the only parts not affected. In unfavorable cases the patient dies from impeded respiration and apoplexy, in consequence of the compression exercised on the air-tubes and jugular veins by the swelling." (p. 2.)

2. *Gases may be developed in the body in consequence of decomposition, fermentation, and putrefaction.* In accordance with the laws of organic chemistry, most animal and vegetable bodies undergo decomposition at the temperature of the human body, and in the presence of moisture, even when air is excluded. Gases are thus developed, which vary in accordance with the putrefying substances that give origin to them. Non-nitrogenous substances yield carbonic acid, carburetted hydrogen, and hydrogen; nitrogenous matters yield ammonia in addition to carbonic acid, and if sulphur and phosphorus are present, sulphuretted and phosphoretted hydrogen, and hydrosulphate of ammonia are also developed. Gas is thus developed in the human body from two distinct sources,—from food in the intestinal canal, in the act of decomposition, and from the decomposition of the tissues of the body itself. The gases produced in the intestinal canal occasionally permeate through its walls into the peritoneal cavity. The slate-gray colour of the surface of the spleen and liver frequently observed in *post-mortem* examinations is due to the action of sulphuretted hydrogen or hydrosulphate of ammonia which must have escaped in this manner.

This chapter concludes with a few observations on the disputed question —*whether different parts of the body can actually secrete gas.* On this point the author offers no decided opinion.

Dropsies. The second chapter is devoted to the consideration of dropsies, which are divided by our author as follows:—1. *Serous dropsy,* or that in which the fluid is identical in its qualitative composition with the serum of the blood. 2. *Fibrinous dropsy,* or that which contains dissolved fibrin, and in its chemical composition resembles the liquor sanguinis. 3. *False dropsy,* in which the fluid differs essentially in its chemical composition from either of the preceding forms.

*Serous dropsy.* Dropsy in which the effused fluid corresponds with the serum of the blood is by far the most common of any of the above forms. Most cases of ascites, hydrothorax, hydrocele, anasarca, and œdema belong to this division, as likewise do the fluids of pemphigus, blisters, &c. Vogel gives a good sketch of the chemistry of the fluids effused in the form of dropsy; as however this subject is fully discussed in the chapter "on the fluid products of disease," in Dr. Day's edition of Simon, we must refer our Sydenhamic—that is, we hope, *all* our—readers to that work for information on the point. Then comes a section on the *causes and mode of origin of serous dropsy,* in which it is traced to a "want of balance between the porosity of the venous walls and the specific gravity of the blood contained therein; that is to say, it occurs when the venous walls become more porous, or the blood lighter, or more aqueous than in the normal condition." In either case there is an increased transudation of serum through the walls of these vessels. This is the manner in which local dropsy invariably occurs where individual veins are compressed or in any way obstructed. It is thus that pressure of the impregnated uterus causes œdema of the feet, and pressure on the *vena portæ* or *vena cava ascendens* produces ascites and œdema of the lower part of the body.

Although there is undoubted evidence that the blood under the influence of strong pressure gives origin to the effusion of dropsical fluids, yet we must not regard the process as a merely mechanical one. Why does not the fibrin which is dissolved in the liquor sanguinis enter into the dropsical fluid? And why, as a general rule, is it that these effusions contain as large an amount of salts, and more water, but less albumen than the serum of the blood? The difficulty of answering these questions shows us that the process is more complicated than at first sight it seems to be. A more accurate acquaintance with the relations of endosmosis than we at present possess is requisite to explain these points in a satisfactory manner.

*Fibrinous dropsy* is regarded by our author as being of more frequent occurrence than the serous form, although it has seldom been described, and has never hitherto received a special name. In its chemical composition the effused fluid is identical with the liquor sanguinis; it is serum with dissolved fibrin. Vogel communicates seven analyses of fibrinous effusions, viz. one by Quevenne of the fluid of empyema, three by himself and two by Scherer of similar fluids, and one by Schwann of the fluid of ascites. Scherer's two analyses are recorded in p. 494 of the second volume of the English edition of Simon, which likewise contains analyses of similar fluids by Simon and Heller. In Schwann's case we cannot help thinking that there must have been some error in the chemical manipulation, the fibrin amounting to no less than 83 in 1000 parts of fluid, or rather more than twenty-four times as much fibrin as occurs in the liquor sanguinis. This fibrinous effusion is attributed by our author to the permeation of liquor sanguinis through the walls of the capillaries. In favour of this view he urges—1st. That the walls of the capillaries are much thinner and more delicate than those of the veins, and that the product of endosmosis will naturally be more concentrated and richer in solid constituents than in the latter case. 2d. That as serous dropsy is associated with dilatation of the veins and attenuation of their walls, so we learn from microscopic examination of the capillary system that a dilatation of those vessels and an attenuated condition of their walls precedes and is associated with the occurrence of the fibrinous fluid either in the parenchyma of an organ or in a cavity.

The fibrinous fluid is capable of organization, which is always effected at the expense of the fibrin contained in it. As far as development is concerned, it is indifferent whether the fibrin has coagulated within the body, or whether it has remained fluid, as in either case it acts equally well as a cytoblastema. There may be produced from it the most varied forms of tissue, either normal—as cellular tissue, simple muscular fibre, cartilage, bone, vessel, or nerve; or pathological—as pus, granular cells, cancer, tubercle, &c. Through this capacity for organization, fibrinous dropsy becomes the common source of a great variety of morbid growths.

*False dropsies* are made to include the cases of dropsy of the kidneys, gall-bladder, lachrymal sac, &c., which are common in the writings of the older pathologists. They are dependent on a closure of the duct of the secreting organ, and a consequent accumulation of the secretion.

PATHOLOGICAL RELATIONS OF THE BLOOD. Deviations in its physical characters, such as are directly obvious to the senses, are first considered; and, secondly, those of a chemical nature, which frequently require complicated processes for their detection.

Amongst the *physical changes*, the first in order is *change in colour.* After noticing the colour of the blood in cases of cyanosis and scurvy, our author remarks that he has "sometimes noticed venous blood of a bright red colour, with a shade of blue, much the tint that is developed on treating uric acid with nitric acid and ammonia. Thus, in the dissection of an arthritic subject, the blood of the renal veins presented this tint." The variations in colour are, however, not sufficiently constant to permit of our always finding the same tint in the same disease, as may be clearly seen by the subjoined table from the recent monograph of Popp* on the Blood in various Diseases:

* Untersuchungen über die Beschaffenheit des menschlichen Blutes in verschiedenen Krankheiten, von Dr. Karl Popp. Leipzig, 1845.

In 133 cases the colour of the blood was observed to deviate from the normal tint 41 times

| | | Ordinary colour. | Very bright red. | Very dark brownish red. | Bluish red. |
|---|---|---|---|---|---|
| In 8 | cases of simple plethora | 5 | 0 | 3 | 0 |
| 2 | pregnancy | 2 | 0 | 0 | 0 |
| 4 | irritation of the brain and spinal cord | 2 | 1 | 1 | 0 |
| 3 | epilepsy | 0 | 2 | 1 | 0 |
| 2 | puerperal convulsions | 0 | 2 | 0 | 0 |
| 2 | hemiplegia | 2 | 0 | 0 | 0 |
| 1 | poisoning by lead | 0 | 0 | 1 | 0 |
| 3 | hypertrophy of the heart | 3 | 0 | 0 | 0 |
| 3 | simple dilatation of the heart | 1 | 0 | 2 | 0 |
| 2 | inflammation of the brain and spinal cord | 2 | 0 | 0 | 0 |
| 1 | inflammation of the brain | 1 | 0 | 0 | 0 |
| 31 | pneumonia | 25 | 5 | 0 | 1 |
| 6 | bronchitis | 5 | 0 | 1 | 0 |
| 1 | metritis | 1 | 0 | 0 | 0 |
| 1 | inflammation of the eye | 1 | 0 | 0 | 0 |
| 1 | erysipelas | 1 | 0 | 0 | 0 |
| 12 | febrile and acute rheumatism | 8 | 3 | 1 | 0 |
| 3 | simple rheumatic fever | 3 | 0 | 0 | 0 |
| 10 | typhus | 2 | 3 | 0 | 5 |
| 2 | scarlatina | 1 | 0 | 1 | 0 |
| 24 | phthisis | 19 | 3 | 2 | 0 |
| 1 | chlorosis, with dilatation of the heart | 0 | 1 | 0 | 0 |
| 1 | scirrhus | 0 | 1 | 0 | 0 |
| 6 | Bright's disease | 5 | 0 | 1 | 0 |

The serum, which in the normal state is clear, is sometimes opaque and of a milk-white appearance. This may depend on various causes. Firstly, on a large number of microscopic fat-globules; secondly, on the presence of a considerable quantity of minute granules of coagulated fibrin; and, thirdly (very possibly), on the formation of a free acid in the blood, whereby a portion of the albuminate of soda becomes decomposed, and albumen, in a finely granular state, is separated. The turbidity dependent on the presence of fat is sometimes observed in perfectly healthy persons, shortly after partaking of an abundant meal, when the blood is receiving a large quantity of chyle. Turbidity, arising from granules of coagulated fibrin, has been observed by Scherer in a pregnant woman with *bronchitis tuberculosa,* in a leuco-phlegmatic person suffering from attacks of vertigo, and in a spirit-drinker with cerebral congestion; by Simon in a man with Bright's disease; and by Zimmerman in several morbid affections.

Deviations in the *consistence, mode of coagulating, odour,* and *taste* of the blood are then considered, and the section concludes with a few remarks on *changes in the corpuscles.* The author has carefully collected all that is known on these points, but has contributed nothing original.

We now arrive at one of the most important subjects in the whole work, and one to which we have already devoted considerable attention in this Journal (Vol. XVII, pp. 136 and 427, and Vol. XX, p. 76); namely, *deviations in the chemical composition of the blood.* Since the publication of Andral's two memoirs in 1840 and 1841, and the works on animal chemistry reviewed in Vol. XVII, the contributions to the chemistry of healthy and diseased blood have been almost overwhelming. Simon's Chemistry, which was published (at Berlin) in 1842, contained, in addition to thirty-two of his own analyses of diseased blood, a full account of all that was then known and of all that had been previously done on the subject. The English editor has added to these the whole pith of the elaborate memoir of MM. Becquerel and Rodier, besides numerous recent analyses by Heller, Scherer, Rinskoff, Herberger, and others, and, in fact, has rendered the chapter on the blood a nearly perfect monograph on the subject up to the commencement of the present year. Although by no means so complete in its various bearings, Vogel's chapter on the pathological chemistry of the blood is still worthy of an attentive perusal by such of our readers as are familiar with the German language, although it contains little in the way of novelty requiring especial notice. His arrangement is excellent; and the following scheme will show that scarcely any important point in the chemistry of the blood is overlooked. The various morbid changes are divided into the two following groups:

I. Those in which any of the normal constituents of the blood are increased or diminished.

*a* An increase or diminution in the amount of fibrin.
*b* An increase or diminution in the amount of blood-corpuscles.
*c* An increase or diminution in the amount of water.
*d* An increase or diminution in the amount of albumen in the serum.
*e* An increase or diminution in the amount of salts.
*f* An increase in the amount of urea.

II. Those in which abnormal constituents are present in the blood.

*a* Free lactic acid.
*b* Carbonate of ammonia.
*c* A substance precipitable by acetic acid, resembling pyin.
*d* Sugar.
*e* Bile-pigment.

In addition to these, he mentions pus-corpuscles and entozoa as foreign organisms to be detected by the microscope rather than by chemical analysis.

In connexion with the determination of the blood-corpuscles by Figuier's method of analysis, which consists in retaining them on a filter after treating defibrinated blood with double its volume of a solution of sulphate of soda of spec. grav. 1·130, (see Day's Edition of Simon, vol. i, p. 190,) Vogel observes that he has always found the filtered solution of a somewhat red tint—a sign that the colouring matter of the blood is not perfectly insoluble in a solution of sulphate of soda. On the whole, however, he seems to approve of the process.

With regard to the abnormal constituents, free lactic acid has been found by Scherer in blood taken from the dead body in cases of puerperal fever and phlebitis, and on several occasions by Vogel, in the blood after death from miliary fever and rheumatism. We regret that no decisive proof of the true nature of the acid is given, as evidence for or against the occurrence of lactic acid in the animal body is just now of considerable importance. Enderlin, and indeed the whole Giessen school, deny that lactic acid can be formed in the human body; and we have the evidence of Pettinkofer that a zinc-salt may be obtained from the urine, resembling in all physical characters lactate of zinc, but differing in the circumstance of the acid containing a considerable amount of nitrogen. While on the other hand, Berzelius, Pelouze, Gobley, and Mulder as strenuously contend that, free or in combination, it occurs in almost every fluid of the body.

Carbonate of ammonia was found by Scherer in blood taken from the arm of a patient with typhus; and the substance resembling pyin was found by the same chemist in the blood of a woman who had died from metro-peritonitis. In his observations on the occurrence of bile-pigment in the blood, our author forgets to mention that the essential matter of bile (biliary resin, bilin, or choleic acid) has been detected by Orfila, Collard de Martigny, Clarion, Pettinkoffer, &c.

The remainder of the chapter is devoted to the consideration of *changes in the quantity of the blood, extravasation of blood,* and *the solution of the hæmatin and the consequent infiltration of the tissues.* These are all fully discussed and admirably illustrated by the Atlas.

We have now arrived at Chapter IV, which occupies no less than two hundred and eighty-three pages, and is devoted to the subject of morbid products and formations.

MORBID PRODUCTS. The following extract "on the development of morbid organic growths" will serve as a fair illustration of our author's plain, straightforward mode of handling his subject:

"It is dependent on laws essentially different from those of mere chemistry. This difference even exhibits itself in the formative material—the cytoblastema; which is most commonly fluid, but may, however, be solid. If solid, it must of necessity be amorphous; in fact, the only solid blastema for morbid products which has yet been observed is coagulated fibrin in an amorphous condition and infiltrated with water, such, for instance, as occurs in inflammatory exudation. But even this blastema was originally fluid, and only became solid through the coagulation of the fibrin. No other coagulated protein-compound, neither albumen, casein, nor globulin, has ever yet been observed to act as a cytoblastema. In the formation of morbid products from a mother-liquid, a solid condition of the plasma has never been observed to occur.

"As in the solid cytoblastema coagulated fibrin seems to act the leading part, so in the fluid cytoblastema dissolved fibrin appears of equally essential importance. This point is, however, of such importance as to require an accurate investigation. It is possible, in many cases, to isolate the fluid blastema of morbid products, and when it occurs in sufficient quantity to institute a chemical examination of it; as, for instance, in cases of exudation into serous cavities, or of a blister under the cuticle. In these cases we find it composed of water, fluid albumen and fibrin, fat, extractive matters, and various salts. That aqueous solutions of salts and extractive matters cannot of themselves alone act the part of a blastema to organized formations is beyond all dispute. They may, indeed, discharge the office of a mother-liquid; in many cases they may even enter into definite forms, as calcareous salts into bone, and chloride of sodium (according to Lehmann) into cartilage; but organized products can only be developed in them when a fibrinous fluid is also present, as, for instance, when exudation takes place into the uropoietic viscera or intestinal canal.

"The same is the case with regard to the fats, some of which admit of deposition in a crystalline form (as cholesterin in gall-stones), and may likewise enter as constituents into organized formations, but none of which, either alone or associated with salts and extractive matters, can act as a cytoblastema; at least, up to the present time no certain case of the kind has ever been observed. There remain, then, as the essential constituents of the blastema only the above protein-compounds, which, however, never occur alone in the body, but always in combination with the above-named substances. But not all even of these are capable of higher development. Fluids which, in addition to the other constituents, contain no other protein-compound than dissolved albumen, never appear to assume the functions of a cytoblastema. Common as albuminous dropsical effusions are, they never give rise to organized forms, unless they also contain fibrin. I have made a very large number of observations on this subject, and have never met with a single exception to the above law. Moreover, a fluid containing casein as its sole protein-compound, seems to be incapable of acting as a cytoblastema. In the milk, for instance, as long as it contains mere casein, we never observe pathological formations, for the so-called *corps granuleux* are dependent on the normal development of milk; but if fibrin becomes mixed with it, morbid formations, as, for instance, pus-corpuscles, are produced. On the other hand, in all fluids which we regard as blastemata for morbid products, fibrin has been invariably detected. Hence we must regard it as the necessary and, apparently, the only essential constituent of the blastema. This law respecting the necessity of the presence of fibrin in these cases, which I have seen to hold good in several hundred instances, without a single exception, is not at all in accordance with the course of normal development. Thus the egg—the prototype of all formative fluids—contains no fibrin, its place being apparently supplied by albumen. There may probably be some connexion between this fact and the observation of Mulder, that the albumen of the egg contains one atom less of sulphur than the albumen of the blood, and that, consequently, in its ultimate composition it is identical with fibrin. In the nutrition of the perfect organism, the general nutrient fluid—the modified blood-plasma permeating the walls of the vessels—acts as the general cytoblastema for all new formations. Whether the fibrin is the only essential formative material, or whether the albumen likewise takes part in development, is a question which cannot be answered with the same degree of certainty as in the case of morbid products, since the normal fluid of nutrition can never be obtained free from extraneous constitutents in sufficient quantity to admit of exact analysis." (pp. 79-81.)

This is succeeded by a sketch of the cellular theory which has been so frequently brought before the notice of our readers that, although Vogel has introduced a considerable amount of original matter, we shall pass on to the consideration of special morbid products. We must not, however, omit to mention, that the cell-theory is excellently illustrated in Plate 1 of the Atlas, which, in addition to a diagram of a cell on a large scale, contains figures of the cells of carcinoma, tubercle, pigment, &c.—in all, twenty figures.

*Fluid Products. Pus.* The first of the morbid products specially considered is *pus,* and we have no hesitation in stating, that in the thirty pages devoted to this subject every fact of importance to the pathologist will be found recorded. The physical characters of normal pus (*pus bonum et laudabile*) are briefly noticed, and we then arrive at the most perfect description of the pus-corpuscle that has ever yet fallen under our notice. After a description of its size, form, and general appearance, we come to the history of its structure, which we shall give at length:

"The corpuscles of normal pus are organized forms, for the most part of a cellular nature, with a nucleus, cell-wall, and contents.

"The cellular structure with a decided nucleus is only apparent in unchanged corpuscles when the cell-wall is very transparent and delicate. In the majority of cases the nucleus is covered by a granulated opaque cell-wall, and does not become visible till the latter is dissolved or rendered transparent by acetic acid.

"In other cases in which the development of the pus-corpuscle is imperfect, we see only the nucleus and no cell-wall.

"The *nucleus* does not lie in the centre of the pus-corpuscle, but is situated eccentrically, and is usually attached to the inner surface of the cell-wall. We may convince ourselves that this is the case by allowing pus-corpuscles to float and rotate under the microscope. It is only the larger nuclei that form an exception to this rule, for they are occasionally so large as to occupy the whole space of the pus-cell. The nucleus of the pus-corpuscle presents many peculiarities, and is so different from other nuclei as to require a somewhat careful consideration.

"In other cells the nucleus is a simple body; but in the pus-corpuscle this is not always, nor indeed generally, the case. It is usually composed of from two to five granules, forming a composite nucleus. Sometimes, on treating fresh corpuscles with acetic acid or with a solution of common salt, we can observe a single nucleus indented like a trefoil leaf, or cloven into two, three, or four smaller nuclei. But it is not every nucleus that undergoes this change; in some cases it appears to resist the action of these reagents. The large corpuscles, with a diameter varying from the 100th to the 80th of a line, exhibit several (two, three, or four) such nuclei, each of which is composed of smaller bodies, insoluble in acetic acid.

"The corpuscles forming the nucleus, when, by the addition of acetic acid, they are clearly brought before us, present various forms; sometimes (generally in good pus) they are elliptic, and present an excavated cup-like form, resembling fresh blood-corpuscles; sometimes, however, they present a spherical or oval appearance. In some cases they are distinct from each other, even lying in different parts of the cell-cavity; but they are more frequently in apposition, and connected together.

"This composite character of the nucleus (as revealed to us by the action of acetic acid) is highly characteristic of normal pus. The only other cases in which it occurs are in young gland-cells, and in the most recent layers of pavement-epithelium; but here they are not so general as in pus. Hence it follows that the size of the nucleus is liable to great variations—from being entirely absent, it may fill the whole cell. Its usual limits are from the 800th to the 400th of a line. Nucleoli are but rarely found in the nuclei of pus-corpuscles.

"The *cell-wall* varies in thickness, and surrounds the nucleus more or less closely. In very delicate and young corpuscles it is extremely thin, smooth, membranous, and transparent; in older and certain peculiar sorts of pus, it is thick, tough, opaque, and studded with minute granules. In many cases the corpuscles have no distinct cell-wall, consisting merely of a nucleus and an irregular deposition around it, without any clearly defined outline, as may be shown, not merely by microscopic investigation, but by its relations towards endosmosis. This is especially the case with young, imperfectly-formed corpuscles.

"Moreover, there are many differences in the *contents of the cell.* In pus-corpuscles with a single nucleus, a well-marked membranous cell-wall, and consequently a cavity between them, we often find no solid body in the cell-cavity except the nucleus: the contents must therefore be fluid, and are doubtless identical with the serum of pus. In other cases, in addition to the nucleus, granular contents, with independent chemical relations, are observed. Sometimes the contents seem so thoroughly fused into the cell-wall, that the two form only a single substance—a solid, but soft mass, in which the nucleus is imbedded." (pp.107-10.)

The endosmotic and chemical relations of the pus-corpuscle are then considered, and the individuality of the following parts of that organism establish:

1. The *substance of the capsule,* which is soluble in solutions of the caustic alkalies and their carbonates, of borax, and for the most part in saline solutions, as those of muriate of ammonia, nitrate of potash, &c.; in part also in acetic acid. It forms the wall and a portion of the contents of the cell, and is doubtless a protein-compound, very similar to and probably identical with that modification of albumen which is precipitable by water, and is again dissolved by the addition of neutral salts or acetic acid.

2. The *substance of the nucleus,* which is insoluble in acetic acid, swells in saline solutions, and dissolves in solutions of borax, of the caustic alkalies, and their carbonates. This likewise is a protein-compound, but its exact nature is doubtful.

3. The substance of which the *minute molecules* consist, which remain undissolved on treating pus-corpuscles with solutions of the caustic alkalies or of borax. Lehmann and Messerschmidt regard this as a protein-compound analogous to keratin (or horny matter), and in many cases this seems to be true. Sometimes, however, these molecules consist of fat; they are then soluble in ether, and if the pus-corpuscles are boiled in that menstruum previously to the addition of the alkali, these molecules do not make their appearance.

Many kinds of normal pus contain nothing in suspension besides the regular corpuscles; others, on the contrary, contain minute roundish molecules, often in a very considerable quantity.* They are always very minute, for the most part less than the 1000th of a line in diameter, and swim (either alone or in heaps) in the serum and between the pus-corpuscles, which are frequently studded with them. These molecules are sometimes protein-compounds,—probably abortive pus-corpuscles; in other cases they consist of fat.

Finally, there are sometimes infusoria present, minute *monades* and *vibriones,* especially in pus from foul ulcers. By feeding them with carmine, Vogel was sometimes able to bring into view the minute specks representing their stomachs.

Moreover, we sometimes find accidental ingredients, as for instance epithelium-cells, fragments of epidermis, crystals of cholesterin or of ammoniaco-magnesian phosphate, or flocculi of amorphous or semi-organized fibrinous exudations. These can be easily detected by the microscope.

In his observations on the serum of pus, the author seems to attach little importance to the substance described by Güterbock under the name of *pyin,* and regarded by that chemist as characteristic of pus. Vogel observes that it is much more commonly found in bad than in good pus; and further, that it occurs in other morbid products, as for instance in carcinoma. After several chemical analyses of pus, we arrive at the important subject of *its formation,* to the consideration of which nearly six pages are devoted. The two following points may be regarded as fully established: 1. Pus-corpuscles cannot be formed from albumen alone. 2. When the full development of the pus-corpusles is completed, the fibrin of the plasma is consumed, and the remaining serum resembles the serum of the blood, or the fluid of serous dropsy.

*Diagnosis of normal pus.* The following remarks on this subject are well worthy of attention.

"The recognition of this morbid fluid is apparently so easy that any one after once seeing it and observing the above physical properties would trust himself to distinguish whether or not a fluid were really pus; and yet there are numerous sources of deception. Fluids containing fragments of epithelium in suspension may readily be mistaken for pus, and on examining the body after death we sometimes believe that we have discovered suppuration, when in fact no such morbid process has been going on. The examination of the fluid under the microscope is the best and, indeed, the only certain means of guarding against such deceptions. If that instrument reveals the presence of normal pus-corpuscles, and on the addition of acetic acid the characteristic nuclei appear, then we may be assured that we have been examining true pus. As I have repeatedly witnessed such deceptions, I will, by way of warning, mention two cases. A woman died from pleuritis with considerable purulent exudation into the pleural cavity. On examination I likewise found in the pelvis of the kidney and the ureter on each side a whitish yellow, thick, creamy fluid, which had all the physical characters of pus, and was mistaken for that fluid by the physicians who were present. As during life there were no symptoms of disease of the kidneys, and as dissection did not reveal any morbid change in those organs, the case was regarded as a demonstration of the resorption of pus, and of its subsequent removal by the kidneys. I examined this assumed pus microscopically, and found in it no trace of pus-corpuscles, but merely broken epithelium from the pelvis of the kidney and ureter.

In another case in which the patient died from peritonitis with exudation, the stomach and upper part of the intestinal canal were perfectly free from remnants of food and chyle, but contained a large quantity of a thick yellow fluid, which was mistaken for pus. In this case also the microscope showed that no pus-corpuscles were present, and that the fluid merely contained the broken cylindrical epithelium of the intestinal canal." (pp. 121-2.)

We fully agree with Vogel, that the microscope is of far more service in the detection of pus than all the so-termed pus-tests; further, it enables us not merely to distinguish pus from mucus, broken epithelium, blood-corpuscles, &c., but likewise to determine approximately the amount of these different substances, which chemical analysis has never succeeded in doing. The characters of Gluge's *compound inflammatory globule* are then noticed, and as we believe that very incorrect ideas are frequently maintained on this subject, and that too great importance is often attached to its occurrence, we shall devote a few lines to Vogel's description of it. Vogel objects to the term *inflammatory globule,* because its connexion with the process of inflammation is not more intimate than that of various other organized formations occurring in exudations; and further, because it is found under conditions in which there is very little probability of its resulting from inflammation—as for instance, in cysts in the thyroid gland. He prefers the term *granular cells.* When these granular cells occur in a perfect condition, they vary in diameter from the 200th to the 80th of a line; some are perfectly round, others oblong, irregular, and even angular. They appear to be agglomerations of minute granules, varying in diameter from the 800th to the 1000th of a line. The following is their mode of formation, as observed by Vogel in inflamed lungs. Cells with a nucleus and nucleolus, differing from pus-corpuscles in their larger size and in having a simple nucleus, are formed in the exudation. These become gradually filled with minute granules, which at first, when only few in number, readily admit of the nucleus being seen; subsequently, however, they conceal it, and the originally smooth cell-membrane becomes rugged, and the granular cell appears as a spherical agglomeration of molecules. Subsequently, the cell-wall appears to vanish, the inclosed granules separate and form irregular heaps, while each granular cell undergoes in miniature the identical process which a mass of coagulated fibrin undergoes in its conversion into pus-corpuscles. This is regarded by Vogel as their usual mode of development; he believes, however, that occasionally the process may be reversed,—that isolated elementary granules are first produced, which collect in groups, and are finally invested with a cell-membrane. Numerous varieties of pus-corpuscles and granular cells are delineated in Plate III of the 'Icones.'

*Solid products.* The solid morbid products next claim our attention. Before proceeding to the consideration of the more strictly morbid textures we have a section extending over upwards of thirty pages, on the regeneration of normal tissues. There are successively considered the epigenesis of areolar tissue (*Bindegewebe*), of blood and vessels, of epithelium and epidermis, of fat and adipose tissue, of muscle, of elastic tissue, of granular pigment, of nervous tissue, and, finally, of cartilaginous and osseous tissues. The section is intended as an introduction to the important subject of *tumours,* which occupies the succeeding 128 pages.

In a histological point of view, tumours may be arranged in two great divisions. In the first, we place those whose histologic elements are identical with those of the normal body, and, further, which when once formed discharge the same duties as the other parts of the body, undergoing metamorphosis of tissue, and receiving their nourishment like a normal portion of the frame—*homologous, non-malignant tumours.*

In the second division we place those whose elements differ histologically from those of the normal body, and which from their nature give way, soften, and destroy the adjacent parts—*heterologous, malignant tumours.*

*Non-malignant tumours* are subdivided by Vogel into—

1. Tumours consisting principally of vessels—vascular tumours.

As a good account of these growths may be found in one of the recent volumes of the Sydenham Society (Swaine's edition of Hasse's 'Pathological Anatomy,' p. 102), we proceed without comment to

2. Tumours consisting principally of fatty tissue—adipose tumours. A true fatty tumour may, according to Vogel, become converted

α. Into general infiltration of fat (*polysarcia, obesitas*) by local hypertrophy of the adipose tissue.

β. Into fibrous tumour by the addition of areolar tissue.

γ. Into encysted tumour by the formation of a cyst around it.

In relation to diagnosis, it must be remembered that many forms of malignant tumour (encephaloid) in their physical characters closely resemble fatty tumours, and can only be distinguished from them by microscopic examination.

3. Tumours consisting principally of fibrous tissue—fibrous tumours.

The following experiment throws some light on the origin of these tumours. Vogel injected several ounces of a solution of hydrosulphate of ammonia into the abdominal cavity of a large dog, and immediately closed up the orifice. At the expiration of twenty-four hours he was killed, and on examination there was found an amorphous exudation of coagulated fibrin on several parts of the intestinal canal under the peritoneum, and blood was extravasated between the muscular and serous coats. On the anterior surface of the stomach there was a coagulum of blood, as large as a hazel-nut, surrounded by a thick layer of fibrin, and firmly attached to the outer wall of the stomach. "I am firmly convinced," adds Vogel, "that this coagulum would in time have been converted into a fibrous tumour, if the animal had not been killed. I have had frequent opportunities of making similar observations. This appears to illustrate the probable origin of fibrous tumours in man—at least of such as occur in the stomach, the intestinal canal, and more especially in the uterus, where there are more frequent opportunities for the formation of coagula."

4. Tumours in which cartilage predominates—cartilaginous tumours.

5. Tumours in which osseous tissue predominates—osseous tumours.

6. Tumours consisting in whole or in part of dark pigment—melanotic tumours. The pigment entering into these tumours is by no means uniform in its characters. In many cases it consists of dark granules inclosed in round or oval cells, sometimes it is modified hæmatin, and it is occasionally composed of granules of sulphuret of iron. The first constitutes true, the two last, false or spurious, melanosis. The pigment is never the sole constituent of these tumours, the other elements being fibrous tissue, a few vessels, and not unfrequently malignant growths, as tubercle, encephaloid, or scirrhus. In true melanosis the colour is brown (of a bistre tint), blackish, or if only a little pigment is present, gray; in the false variety resulting from altered hæmatin, it is blue, violet, or brownish-black; and in that depending on sulphuret of iron it is of a slate-gray, or greenish-black. Melanotic tumours are more frequently observed in the female than the male sex.

7. Tumours containing gelatinous matter—gelatinous tumours; which are described by Vogel as follows:

"In many tumours there occurs a viscid jelly-like substance, partly infiltrated amongst the firm elementary tissues, and partly contained in appropriate spaces or cavities, sometimes in such abundance, and so greatly exceeding the other elements, that the tumours may with great propriety be termed gelatinous. The elements coexisting with gelatine in these tumours are various, generally fibres, vessels, and occasionally cartilage, and most commonly of all, cancer-cells forming gelatinous cancer—*colloid.*

"This substance is always transparent and colourless, sometimes fluid, resembling thickened mucus; at other times resembling half-fluid jelly. Under the microscope it appears completely amorphous, and so perfectly transparent that it is not easy to see it. In six cases in which I have examined it, its physical and chemical characters appeared constant and identical with those of mucus.....Nothing can as yet be stated with certainty respecting the origin of this substance; it arises, however, in all probability, like normal mucus, from modified protein-compounds of the blood." (pp. 204-5.)

8. Tumours inclosed in a true cyst—encysted tumours.

The *malignant tumours* are divided into two leading groups. The former embraces those in which little (or no) organization is apparent, namely:

1. *Typhous deposits.*
2. *Scrofulous deposits.*
3. *Tubercle.*

The second includes the more highly organized secondary formations; namely, *carcinoma,* subdivided into

1. Cellular cancer—encephaloid.
2. Fibrous cancer—scirrhus.
3. Melanotic cancer.
4. Gelatinous cancer—colloid.

We pass over these subjects without comment; the character of the typhous deposit having been noticed in our review of Rokitansky, in Number XXIX, and cancer fully discussed in our recent review of Professor Walshe's 'Treatise.'

The remainder of this lengthy chapter is devoted to the consideration of unorganized morbid products.

The following scheme includes the various substances capable of occurring as deposits in the human body:

1. Protein-compounds.
2. Fats: *a,* cholesterin; *b,* margarin and margaric acid; *c,* olein; *d,* fatty granules of uncertain composition.
3. Uric acid and urates.
4. Salts of lime: *a,* oxalate of lime; *b,* basic phosphate of lime; *c,* carbonate of lime.
5. Ammoniaco-magnesian phosphate.
6. Sulphuret of iron.
7. Bile-pigment.
8. Silica.
9. Various substances of easy solubility, deposited in consequence of evaporation, &c., as chloride of sodium, phosphate of soda, &c.

From one or more of these constituents are formed all the concretions occurring in the human body. These are arranged by Vogel into

1. Such as are produced from the secreted fluids.
2. Such as are formed in the parenchyma of organs.

The subject is completely treated in all its bearings, but contains little with any claim to novelty. The analyses of the concretions are for the most part taken from Simon.

We now arrive at a short chapter of twenty pages on the morbid changes in the physical properties of the tissues and organs. Changes of colour, volume, form, and consistence, are discussed, and a considerable amount of original matter introduced.

After a very brief and somewhat unsatisfactory chapter on the combinations of morbid changes, we come to the consideration of *independent organisms in the human body—parasites.* These are divided into *Epiphytes* and *Parasitic animals.*

The *Epiphytes* are subdivided into (1) vegetations in the human fluids, namely, the *torula cerevisiæ* in vomited fluids and fæcal evacuations, and Goodsir's *sarcina ventriculi;* (2) vegetations on the external skin and its appendages, namely, in *Tinea favosa* (Gruby, Fuchs, Bennett), in the sheath of the hair in *mentagra* (Gruby), within the root of the hair in *Herpes tonsurans* (Gruby, Hebra), and in *Plica polonica* (Gunsburg); and (3) vegetations on the mucous membranes, as, for instance, in the aphthæ of children, in the cicatrices of the mucous membrane after typhus, &c.

*Parasitic animals* are subdivided into infusoria, insects, arachnida, and worms.

The following experiment, in relation to infusoria in the blood, is interesting, although leading to a negative result. Two ounces of water, containing millions of infusoria of the same species (either *monades* or the young of the *cyclidium glaucoma*), were injected into the vein of an adult cat. At the expiration of twenty-three hours not a trace of the infusoria could be observed, and when the animal was killed, two days subsequently, the search was equally unsuccessful. If Siebold's* article on parasites is studied conjointly with the present chapter of Vogel, the reader will acquire an immense amount of information that may in vain be sought for in English or French medical literature.

Congenital changes in the human body are then considered. The causes of malformation are, according to our author, the following:

1. Abnormality of the generative material of one or both parents.

2. Abnormality of the maternal organism, pathological changes in the uterus, fallopian tubes, &c.

3. Diseases and abnormal conditions of the placenta, membranes, or cord.

4. Pathological affections directly affecting the fœtus—diseases and mechanical injuries.

The different forms of malformations are arranged in eight classes, most of which are divided into orders and genera. The difficulty of condensing this subject within the limits of some forty pages will be at once recognized by those who are conversant with the voluminous works of Meckel, Geoffroy St. Hilaire, Otto, Gurlt, Vrolic, &c. The present chapter (although little more than a syllabus) contains infinitely more available matter than the works of any one of the authors just quoted.

The part now before us concludes with a chapter on the changes occurring directly after death. Like all that precedes it, it is well worthy of perusal, but contains no especial novelty either in the way of arrangement or material.

From our previous knowledge of the writings of Vogel, and from the high reputation that he has acquired during the last seven or eight years (and pathological histology is of no older birth), we took up our pen with a decided prepossession in his favour. If we have been slightly disappointed in not finding so many original observations as we expected, that disappointment has been more than counterbalanced by the philosophic spirit pervading the whole work. Unlike Lebert, whose work is noticed in our last Number, he strives not for glory but for truth, and every page shows his desire to render to all men their due. Here we have none of the self-gratulation and assumed superiority for which we were compelled to inflict a moderate castigation on the pseudo-Frenchman; on the contrary, we can lay our hand on several pages in which M. Vogel desires to recant or modify opinions he had previously adopted; and the truthful singleness of mind he displays cannot fail to have its due weight with his readers. In his knowledge of the foreign literature of his subject, we may express our decided conviction that he stands unrivalled. Carswell and Hope, Owen and Liston, Simpson and J. H. Bennett, are but a few of the British names that meet us in his pages. France, Italy, Holland, and Denmark,—all lay open their stores before him, and he gathers as freely from Cruveilhier and St. Hilaire, Cerutti and Bassi, Vrolik and Broers, Hannover and Steenstrup, as from his own countrymen Müller, and Henle, and Gluge.

We have had occasion, in several parts of this review, to notice the

* Wagner's Handwörterbuch, &c., vol ii, pp. 641-92.

Atlas; but if we spoke of it as a mere volume of illustrative plates we should be doing its author a great injustice. Of the 291 figures contained in it, 270 are drawn from nature, and accompanied with copious explanations extending over 128 quarto pages. Of the accuracy of most of the figures we are enabled to speak from experience.

We are glad to learn that this work is likely soon to appear in an English dress, from the competent pen to which we are indebted for the excellent work of Simon, recently issued by the Sydenham Society. We trust that Dr. Day will incorporate in it the most important cases from the 'Icones,' and have it properly illustrated. We do not doubt that a work of this nature, with microscopic delineations of morbid products, would supply a want which has been long experienced in this country.

---

